# Time and Crack Width Dependent Model of Chloride Transportation in Engineered Cementitious Composites (ECC)

**DOI:** 10.3390/ma15165611

**Published:** 2022-08-16

**Authors:** Linglai Bu, Lei Qiao, Renjuan Sun, Wei Lu, Yanhua Guan, Nan Gao, Xinlei Hu, Zhenhuan Li, Lin Wang, Yuhe Tian, Yu Qin

**Affiliations:** 1 School of Qilu Transportation, Shandong University, Jinan 250002, China; 2 Shandong High-Speed Engineering Construction Group Co., Ltd., Jinan 250014, China; 3 Jining Construction Project Quality and Safety Technology Center, Jining 272000, China; 4 Chongqing Luneng Development (Group) Co., Ltd., Chongqing 404100, China

**Keywords:** engineered cementitious composites, surface chloride concentration, apparent chloride ion diffusion coefficient, crack width

## Abstract

This paper aims to develop a chloride transport model of engineered cementitious composites (ECC) that can consider the influence of both exposure time and crack width. ECC specimens with crack widths of 0.1 mm, 0.2 mm and 0.3 mm were soaked into NaCl solution with periods of 30, 60, 90 and 120 days. The free chloride content profile was measured and used for the development of the transport model. Regression analysis was applied to build the time and crack width dependent models of apparent diffusion coefficient and surface chloride content. The results show that the crack width has significant influence on the free chloride concentration profile when it is above 0.2 mm and the time-dependent constant *n* decreases linearly with the crack width. The chloride transport model was obtained by subscribing the models of apparent diffusion coefficient and surface chloride content into the analytical solution of Fick’s second law. The model was further validated with the experimental results, showing a deviation within 20%. The findings of the presented study can enhance the current understanding on the chloride transportation in ECC.

## 1. Introduction

The durability problem of reinforced concrete structures is mainly caused by the corrosion of reinforcement in concrete. Exposed to seawater and a deicing salt environment, reinforced concrete structures are prone to chloride corrosion, which leads to cracking on the concrete cover and a reduction of service life [1,2,3]. Chloride corrosion of concrete structures produces large economic losses every year [4,5,6].

The diffusion of free chloride ions through pores and cracks is the main reason for reinforcement corrosion in concrete structures. An effective way to reduce chloride diffusion is to improve concrete density and cracking behavior. Engineered cementitious composite (ECC) is a highly ductile material reinforced with fibers, which can effectively control the width of cracking [7,8], generally not exceeding 80 μm. Its tensile performance is hundreds of times higher than that of ordinary concrete [9,10,11,12], which makes ECC more resistant to chloride corrosion. Compared with concrete of the same strength, the chloride ion diffusion coefficient of ECC is only about 10–35% of that of the counterpart [13]. ECC can also retain a good resistance to chloride attack after cracking [14,15,16,17]. It has been extensively used in the concrete structures exposed to harsh environment [18].

A great deal of numerical studies focused on the effects of material and environment parameters on the chloride diffusion coefficient and established relevant models. Generally, diffusion of ions through porous media are following Fick’s 2nd law. Yu et al. [19] studied the influence of water-binder ratio, fly ash, and slag contents on chloride diffusion coefficient of concrete and established a predictive model based on regression method. Chidiac et al. [20] developed a resistivity model to quantify the chloride diffusivity of concrete. Lawi al-Sodani et al. [21] investigated the influence of temperature on chloride diffusion in concrete and developed a chloride diffusion model using experimental data. Wang et al. [22] addressed the effects of volume fractions of coarse aggregate and reinforcement on chloride diffusion in concrete and proposed a prediction model in function of them. Chalee et al. [23] proposed a model for the diffusion coefficient of concrete with large amount of fly ash under long-term exposure. An exponential relationship between time and apparent chloride diffusion coefficient was employed by Mangat and Limbachiya [24], with w/cm of 0.45 to 0.65 and fly ash contents of 0% to 50%. The Life—365 model specified that the maximum replacement levels of SF, FA, and GGBFS were limited to 15%, 50%, and 70%, respectively, which accounted for the w/cm, cementing material type, time, and temperature [25]. The effect of cracks on chloride diffusion has not been fully understood. Limited studies [18] on crack influence in chloride diffusion of ECC have been conducted. Zhang et al. [26] investigated the effect of cracks in low shrinkage engineered cementitious composite (LSECC) on chloride penetration. A comprehensive review of the durability of ECC was conducted by Ma et al. [27] and it was indicated that the average crack width should be designed to be <100 μm to maintain high durability. Mustafa Şahmaran et al. [28] performed chloride penetration tests and accelerated aging and elaborated that no significant difference in the effective diffusion coefficient was observed when crack widths were changed between the 10 and 50 µm. Although it is generally believed that the fine cracks can effectively limit the penetration of chloride ions, studies have shown that the resistance of cracked ECC against chloride ingress is mainly governed by the accumulated crack width rather than the maximum one [29,30]. Therefore, influence of a relatively large crack width, typically larger than 0.1 mm, on the chloride penetration should be revealed.

Considering the difficulties on controlling the accumulated crack width of ECC, cracks with widths of 0.1 mm, 0.2 mm, and 0.3 mm were notched on the ECC specimens herein. The specimens were soaked into NaCl solution with periods of 30, 60, 90, and 120 days. The free chloride content profile was measured and used for the development of the transport model based on the Fick’s second law. The influence of exposure time and crack width on the apparent diffusion coefficient and surface chloride content was revealed. The model was validated using the experimental results, showing reasonable agreement for most cases.

## 2. Materials and Methods

### 2.1. Materials and Properties of ECC

The raw materials for ECC consist of P.O. 42.5 Portland cement, Class F fly ash, quartz sand with particle size ranging from 120 to 180 μm, PVA fiber, hydroxypropyl methylcellulose thickener (HPMC), polycarboxylate superplasticizer, and water. The PVA fibers were produced by the Japan Kuraray Company. The mix proportion of ECC used in the experiment is shown in Table 1. The detailed properties of PVC fiber are shown in Table 2.

### 2.2. Specimens and Exposure Conditions

**Figure 1:**
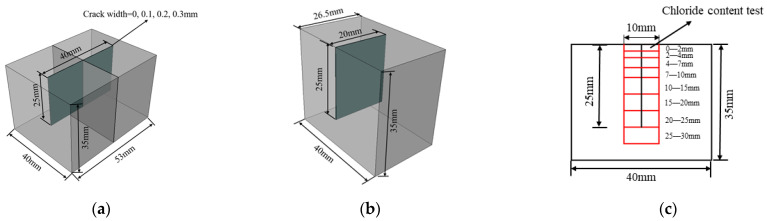
Schematic view of the specimen used for the analysis. (**a**) Specimen used for immersion (**b**) specimen cut in half for the measurements (**c**) slices used for free chloride content test.

Specimens with size of 53 × 40 × 35 mm were used in this study. Before the test, all the specimens were cured in a room with relative humidity of 95% and temperature of 20 ± 2 °C for 28 days. The crack was created by inserting a prefabricated thin steel sheet with a certain depth of 25 mm into the fresh ECC mixture after poured into the molds. It was notched on the middle line of surface with size of 53 × 40 mm which is paralleled to the long side as shown in Figure 1a. It has been confirmed that there is an upper and lower threshold for the crack width affecting the chloride ion erosion of concrete [29,30,31,32]. When the crack width is less than the lower threshold, the chloride ion transport in concrete is almost unaffected by the crack while when the crack width is greater than the upper threshold, the chloride ion erosion along the crack wall is basically the same as the main erosion surface. Based on previous studies [26,33], three pre-crack widths were chosen, i.e., 0.1 mm, 0.2 mm, and 0.3 mm; see Table 3. The cracked surface was exposed to the chloride environment, while the other five faces were coated with epoxy resin. To simulate a chloride concentration similar to the marine environment, 3.5% (mass ratio) NaCl solution was used. Four exposure periods were considered, namely 30, 60, 90, and 120 days. Three specimens were tested for each case under a certain exposure time and the average value was used. In total, 48 specimens were tested.

### 2.3. Free Chloride Content Measurement

Once reaching the targeted exposure period, specimens were taken out and cut as shown in Figure 1b. Then they were sliced and sampled as shown in Figure 1c. The slice was about 10 mm in width and 2 mm in thickness. The depths were set to 0–2 mm, 2–4 mm, 4–7 mm, 7–10 mm, 10–15 mm, 15–20 mm, 20–25 mm, and 25–30 mm along the crack depth direction. The ECC slices were dried and milled. The slices were crushed and ground into a powder and sieved using a 0.3 mm sieve. The collected powder was oven dried at 60 °C for 24 h and used for the free chloride content measurement. In the measurement, 2 g of powder was dissolved in 200 mL distilled water using magnetic stirrers. After 30 min of mixing, the free chloride content test was measured using a NELD-CL420 chloride ion selective electrode.

### 2.4. Analysis of Variance (ANOVA)

Analysis of variance (ANOVA), also known as ‘analysis of variance’ or ‘F-test’, is a statistical method used to test the significance of differences between the means of two or more samples. The ANOVA concluded that the differences between the means of the different treatment groups resulted from the test conditions and random errors.

Differences due to experimental conditions, i.e., treatments, are expressed as the sum of the squared deviations of the means of the variables in each group, denoted as *SST* Differences caused by random errors such as measurement are expressed as the sum of the squared deviations of the variables from the mean of the group, denoted as *SSE*. *SST* and *SSE* are divided by their respective degrees of freedom to obtain *MST* and *MSE*; see Equations (1) and (2).

(1) $$MST=\frac{SST}{q-m}$$

(2) $$MSE=\frac{SSE}{m-1}$$

where *q* is the total number of samples and *m* is the number of groups

The ratio of *MST* to *MSE* forms the F-distribution; see Equation (3). *F* is approximately equal to 1 when all groups of samples are from the same aggregate, and much greater than 1 when each sample is from a different aggregate. The probability of the F-value being greater than a specific value given that the test hypothesis holds can be obtained by consulting the table of F-bound values for ANOVA. The value of *p* is the probability of making an error when the observation is considered valid. In the above assumption, the value of *p* is equal to the stated probability. When *p* is less than 0.05, the two sets of data are considered statistically significant, while *p* is less than 0.01, the two sets of data are considered highly statistically significant.

(3) $$F=\frac{MST}{MSE}$$

where: *F* is ANOVA coefficients; *MST* is the mean sum of squares due to treatment; *MSE* is the mean sum of squares due to error.

## 3. Experimental Results

**Figure 2:**
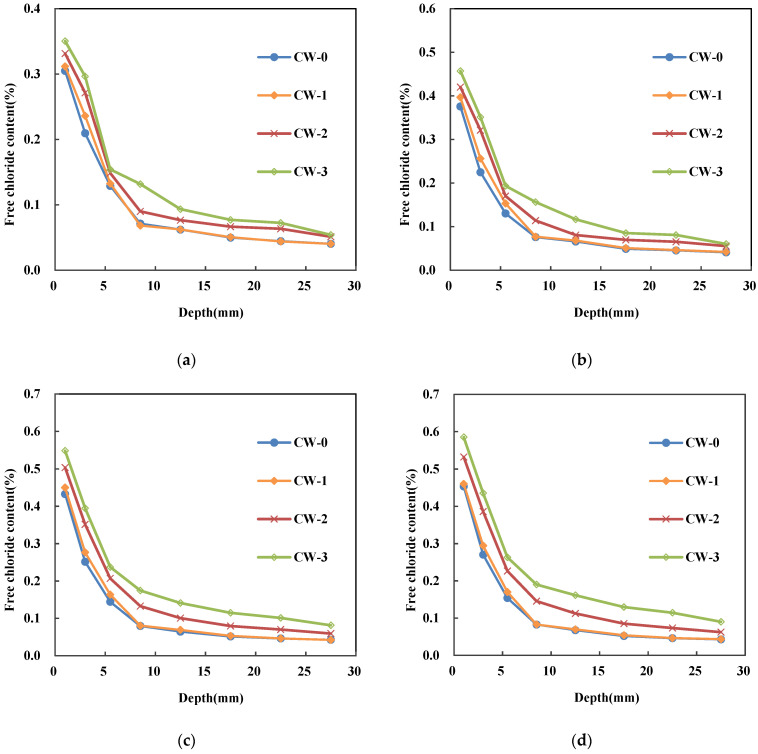
Variation of free chloride content with crack depth. (**a**) 30 days (**b**) 60 days (**c**) 90 days (**d**) 120 days.

The variation of free chloride content profile under different exposure periods is shown in Figure 2. In general, the free chloride content decreases with the depth, and a high rate of reduction is observed when the depth is smaller than 10 mm. Once the depth is above 15 mm, the decline of the free chloride content become quite gentle. For each sample, the free chloride content at the same depth increases with the exposure time. For example, a significant increase of 66.9% can be observed for CW-3 in the depth of 0–2 mm at 120 days compared to that at 30 days. An obvious growth of 46.9% can also be found for CW-3 in the depth of 2–4 mm at 120 days compared to that at 30 days. No significant difference is observed for the free chloride content profile of CW-0 and CW-1. Once the width of crack is above 0.2 mm, a remarkable increment of chloride content is observed. An increase of 8.7% and 14.9% is observed for CW-2 and CW-3 compared to that of CW-0 in the depth of 0–2 mm at 30 days. These increments reach 17.4% and 29.4% respectively at 120 days.

### 3.1. Chloride Transport Model Development

**Figure 3:**
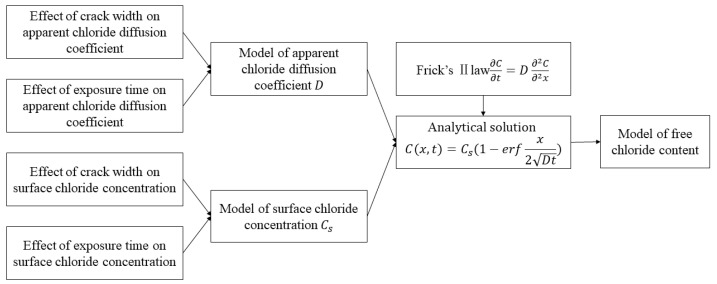
Development of the model of chloride transportation in ECC.

Based on the experimental results, it can be found that there are non-negligible effects of crack width and exposure time on chloride ion transportation in ECC. Regression analysis was used to obtain the effects of two variables, namely exposure time and crack width, on the two key parameters for chloride ion transportation, namely surface chloride concentration and apparent chloride diffusion coefficient. The surface chloride concentration and the apparent chloride ion diffusion coefficient models were built and substituted into the chloride transportation equation based on Fick’s second law and then the chloride transport model was obtained, see Figure 3.

#### 3.1.1. Fundamental of Chloride Transport Models

Chloride transport in cement-based materials is a complex physical and chemical process, and the most common transport mechanisms are diffusion, permeation, migration, and convection [34]. Under long-term immersion, chloride transport is dominated by diffusion. Apparent chloride diffusion coefficient can be used to express the chloride resistance of cement-based materials. When considering this parameter, diffusion theory should be applied. Fick’s second law is commonly used for determining the apparent chloride diffusion coefficient [34,35,36]:

(4) $$\frac{\partial C}{\partial t}=D\frac{\partial^{2}C}{\partial^{2}x}$$

where *C* is the chloride content, *t* is the time, *D* is the chloride diffusion coefficient, and *x* is the spatial coordinate. If the diffusion coefficient is assumed as constant, the equation has an analytical solution as:

(5) $$C\left(x,t\right)=C_{s}\left(1-erf\frac{x}{2\sqrt{Dt}}\right)$$

where *C* (*x*, *t*) is the chloride content (%) at a certain depth *x* (m) and exposure time (s), *C*_s_ the surface chloride concentration, *D* the apparent chloride diffusion coefficient (m^2^/s), erf the Error function. *C*_s_ can be approximated as the chloride content in the depth of 0–2 mm [37]. By fitting the measured chloride penetration profiles using Equation (5), the apparent chloride diffusion coefficient and surface chloride concentration for each case can be determined. However, as it has been stated in the previous studies that both *C*_s_ and *D* varies with the exposure period and crack width [38,39]. Therefore, a model that can be used to consider both exposure time and crack width should be proposed.

#### 3.1.2. Model the Surface Chloride Concentration

**Figure 4:**
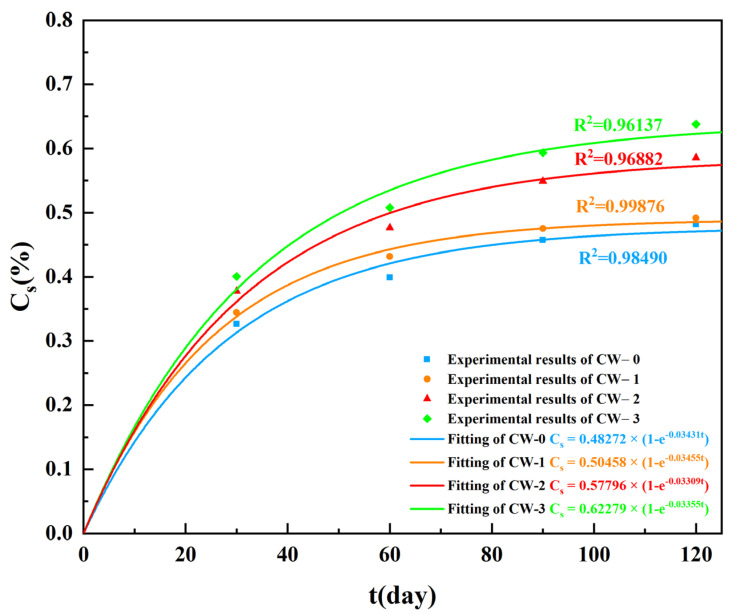
Influence of exposure time on surface chloride concentration.

Figure 4 shows the evolution of surface chloride concentration along the exposure time. Clearly, with the same crack width, the increase rate of *C*_s_ decreases with the time. This can be attributed to the going hydration of cement [40,41,42], pozzolanic reaction of the fly ash [43,44,45], and chloride ions binding [46,47], which densify the pore structure of ECC. The exponential function is generally used to fit the relationship between *C*_s_ and *t* [48]; see Equation (6).

(6) $$C_{s}=a\left(1-e^{-bt}\right)$$

where *a* and *b* are the fitting parameters. Figure 4 shows the fitted results using the least square method. It can be seen that the exponential function shows a quite high determinization coefficient for all cases. The fitted value of b almost remains unchanged and sits in the range of 0.033–0.035. The average value, i.e., 0.03375 is therefore used in the current study.

**Figure 5:**
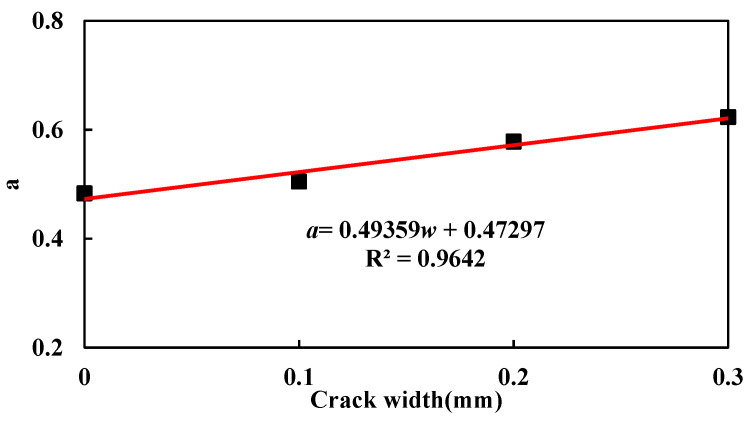
Relationship between *a* and crack width.

Figure 5 shows the relationship between *a* and crack width *w.* A linear function as shown in Equation (7) can be used to properly describe it with a determination coefficient of 0.9642.

(7) a=0.49359w+0.47297

**Figure 6:**
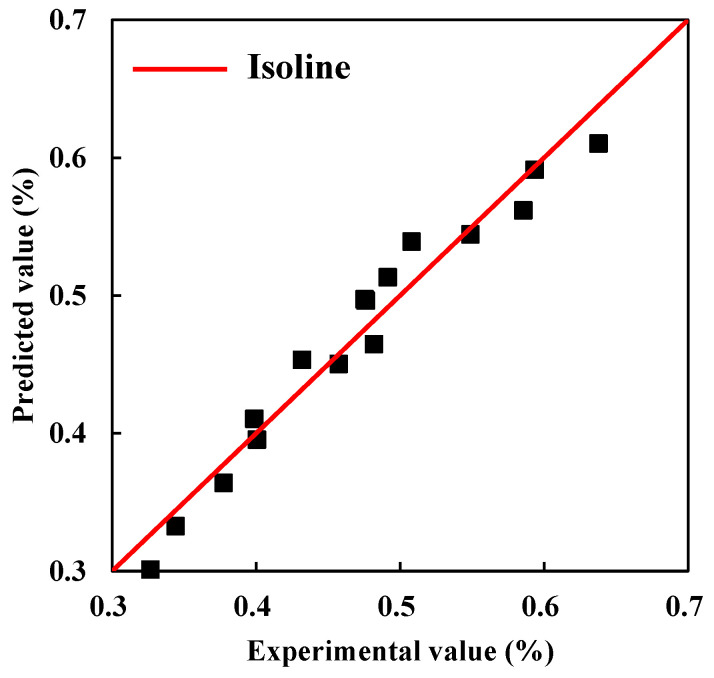
Predicted effect of surface chloride concentration.

The bivariate function as shown in Equation (8) was proposed to consider the combined influence of exposure period and crack width on the surface chloride concentration *C*_s_. The predicted results are compared with the measured values and show high accuracy; see Figure 6.

(8) $$C_{s}=(0.49359w+0.47297)\left(1-e^{-0.03375t}\right)$$

Analysis of variance (ANOVA) using the F-test was conducted to test the significance of the effect of each variable on the surface chloride ion concentration. The model passes the F-test and the value of *p* is less than 0.01 in both the one-way ANOVA and two-way ANOVA, which indicates that both crack width and exposure time have significant effects on surface chloride ion concentration at the 0.01 level; see Table 4.

#### 3.1.3. Model the Apparent Chloride Diffusion Coefficient

**Figure 7:**
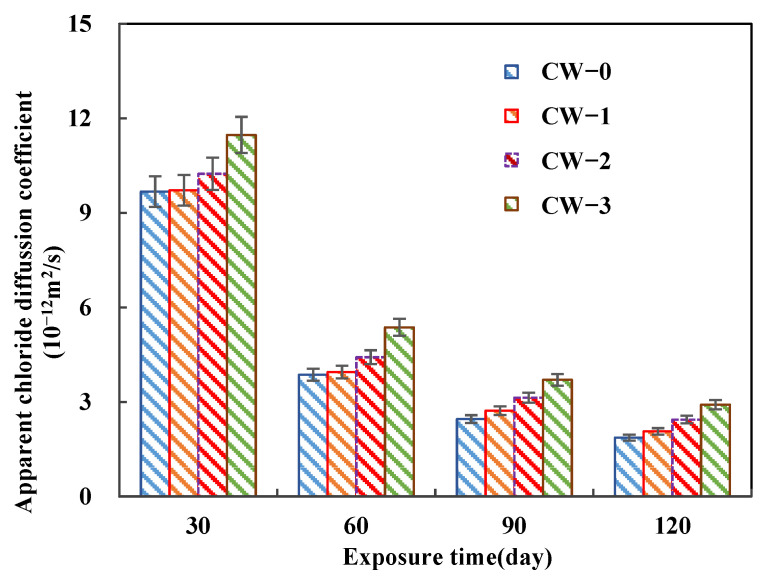
Variations of apparent chloride diffusion coefficient of ECC with different crack widths.

The variation of the determined apparent chloride diffusion coefficients of all cases are shown in Figure 7. Clearly, the chloride diffusion coefficient decreases with the exposure time due to the densification of the microstructure caused by the chloride erosion products and the continuous hydration of the cementitious materials [49]. Furthermore, the decreasing rate decreases with the ongoing exposure. The chloride diffusion coefficient decreases nearly 50% from 30 to 60 days, about 30% from 60 to 90 days, and decreases only 5% from 90 to 120 days. This can be attributed to the fact that, with the exposure period increasing, the number of reactive phases become less.

**Figure 8:**
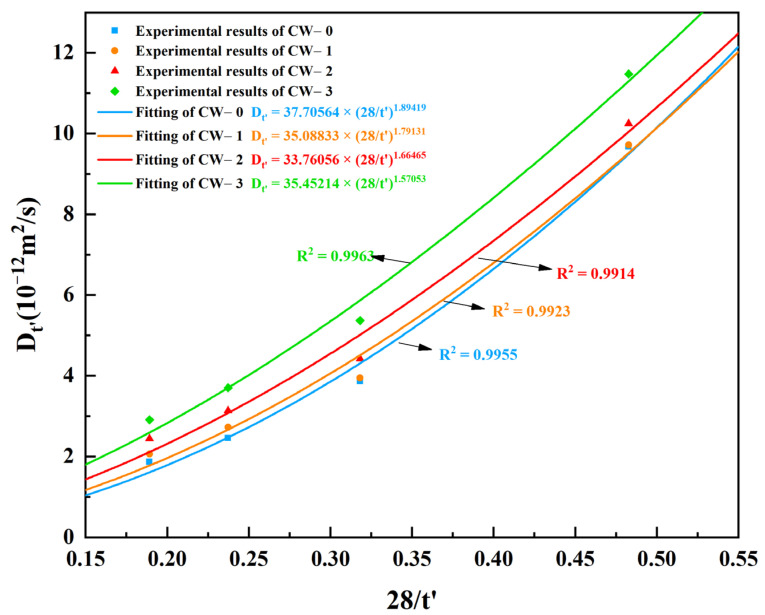
Relationships between the apparent chloride diffusion coefficient and exposure time.

**Figure 9:**
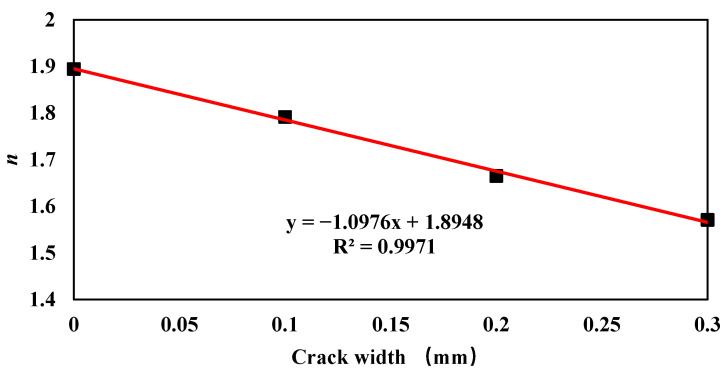
Relationship between *n* and crack width *w*.

The time-dependent apparent chloride diffusion coefficient is often described by Equation (9) [35,50]:

(9) $$D_{t^{\prime }}=D_{t0}{\left(\frac{t^{\prime }}{t_{0}}\right)}^{-n}=D_{t0}{\left(\frac{t_{0}}{t^{\prime }}\right)}^{n}$$

where $D_{t^{\prime }}$ is the apparent chloride ion diffusion coefficient at the concrete age of $t^{\prime }$; $D_{t0}$ is the apparent chloride ion diffusion coefficient at the concrete age of $t_{0}$; *n* is the time-dependent coefficient. In general, *t*_0_ is chosen as 28 days as shown in Equation (10).

(10) $$D_{t^{\prime }}=D_{28}{\left(\frac{28}{t^{\prime }}\right)}^{n}$$

where *D*_28_ is the apparent chloride ion diffusion coefficient at 28 days, which can hardly be measured. Therefore, both *D*_28_ and *n* are fitted from the experimental results. Figure 8 shows the fitted results. Clearly, the crack width does not significantly influence *D*_28_, as at the first beginning of the test, the mainly driving force of the chloride ingress is capillary suction [38]. The average value, i.e., 35.5 × 10^−12^ m/s^2^ was used to represent *D*_28_. Additionally, with the increase of crack width, the value of time-dependent constant *n* decreases gradually. Compared with CW-1, the value of *n* for specimen CW-1, CW-2 and CW-3 decreases by 5.4%, 12.1% and 17.1%, respectively. The relationship between *n* and crack width was fitted using a linear function; see Equation (11). It can be seen from Figure 9 that the fitting shows satisfactory results with a determination coefficient of 0.9971.

(11) n=−1.0976w+1.8948

By substituting Equation (11) into Equation (10), the time dependent apparent chloride diffusion coefficient model considering the effect of crack width can be obtained.

(12) $$D_{t^{\prime }}=D_{28}{\left(\frac{28}{t^{\prime }}\right)}^{-1.0976w+1.8948}$$

**Figure 10:**
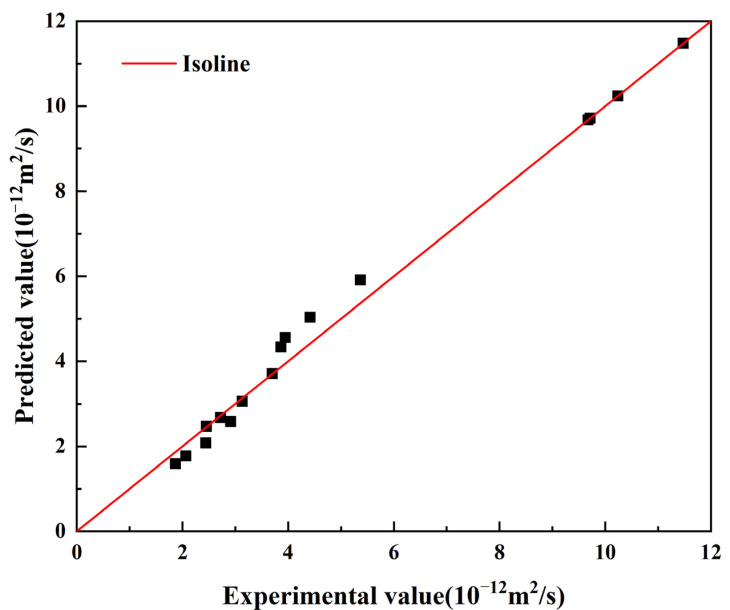
Comparison between the measured and predicted apparent chloride diffusion coefficient.

The predicted apparent chloride ion diffusion coefficients under various conditions were compared with the experimental results and show high accuracy; see Figure 10.

Analysis of variance (ANOVA) using the F-test was conducted to test the significance of the effect of each variable on the apparent chloride diffusion coefficient. The model passed the F-test, and the value of *p* was less than 0.01 in both the one-way ANOVA and two-way ANOVA; see Table 5.

#### 3.1.4. Model Validation

The chloride transportation model considering both the crack width and exposure time was obtained by substituting Equations (8) and (12) into Equation (5).

(13) $$C\left(x,t\right)=(0.49359w+0.47297)\left(1-e^{-0.03375t}\right)\left(1-erf\frac{x}{2\sqrt{D_{28}{\left(\frac{28}{t^{\prime }}\right)}^{-1.0976w+1.8948}t}}\right)$$

**Figure 11:**
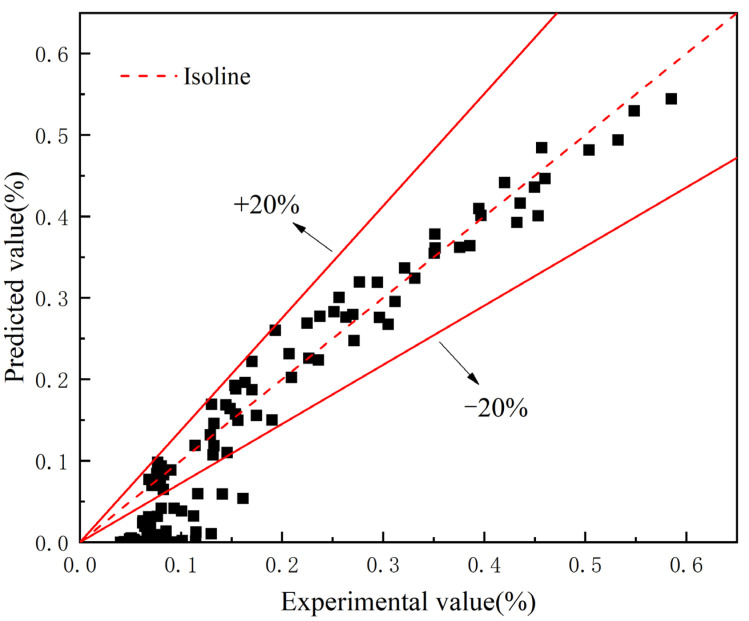
Relative error analysis of the prediction model.

The developed model was then compared with the experimental data. Clearly, almost of the experimental results falls in the 20% intervals. Relative error analysis of the model is shown in Figure 11, where it is found that the free chloride concentration can be well predicted by this model when the chloride concentration is higher than 0.15%. The deviation at the small chloride concentration mainly comes from the location at the large depth of the specimens (typically >22.5 mm). The Fick’s second law assumes an asymptotic line occur when the depth is large enough. This is true when the crack width is smaller than 0.1 mm. However, in terms of crack width of 0.2 mm and 0.3 mm, the measured depth is not large enough to reach the asymptotic boundary. As can be seen from the experimental results shown in Figure 2, there is still a clear downward trend in the free chloride ion content when the depth reaches the 30 mm, which is the maximum in the current study. This leads to the overestimate of the free chloride content in the location larger than 22.5 mm. The location deeper inside the specimen should be measured in the further study to provide sufficient data for the model. Nevertheless, in most cases, the deviation between prediction and experiment is smaller than 20%.

## 4. Conclusions

In this paper, the effects of crack width and exposure time on the apparent chloride ion diffusion coefficient and surface chloride concentration were analyzed. A model considering both crack width and exposure time was developed to simulate the free chloride content concentration profile. The main conclusions were as follows.

The free chloride content decreases with the depth and a high rate of reduction can be noted when the depth is smaller than 10 mm. For each sample, the free chloride content at the same depth improves with the exposure time. The crack width has a significant influence on the free chloride concentration profile when it is above 0.2 mm. The free chloride content for CW-2 and CW-3 was17.4% and 29.4% higher than that of CW-0 in the depth of 0–2 mm respectively at 120 days.

The relationship between surface chloride concentration and exposure period can be expressed by an exponential function. The exponential term is not influenced by the crack width and the constant *a* has a linear relationship with crack width.

The time-dependent apparent chloride diffusion coefficient model can satisfactorily consider the change of apparent chloride diffusion coefficient along hydration time. The time-dependent constant *n* decreases linearly with the crack width.

The validation shows that the developed chloride transport model can effectively consider the coupled influence of crack width and exposure time. In general, the deviation between prediction and experiment is smaller than 20%.

## Figures and Tables

**Table 1:** Mix proportion of ECC (kg/m^3^).

Materials	Cement	Fly Ash	Sand	HPMC	Water	Water Reducer	PVA Fibers	ECC
Quantity	568	682	455	0.5	325	10	26	2066.57

**Table 2:** Properties of PVA fiber.

Tensile Strength (GPa)	Tensile Modulus (GPa)	Diameter (µm)	Length (mm)	Elongation (%)	Density (g/cm^3^)
1.56	41	40	12	6.5	1.3

**Table 3:** Test configurations.

Test Number	Crack Width (mm)	Exposure Period (Days)
CW-0	0	30, 60, 90, 120
CW-1	0.1	
CW-2	0.2	
CW-3	0.3	

Note: CW represents the crack width; CW-1 respresents that the crack width is 0.1 mm.

**Table 4:** F-test results of the proposed model for surface chloride concentration.

	*F*	*p*
Exposure time	73.61717	1.18382 × 10^−6^
Crack width	32.35408	3.76888 × 10^−6^
Proposed model	52.98563	1.62782 × 10^−6^

**Table 5:** F-test results of the model of apparent chloride diffusion coefficient.

	*F*	*p*
Exposure time	1487.09451	1.90548 × 10^−12^
Crack width	44.77743	9.81442 × 10^−6^
Proposed model	765.93597	1.14968 × 10^−11^

## Data Availability

The general data are included in the article. Additional data are available on request.
